# Supplementary material for: Profound Nanoscale Structural and Biomechanical Changes in DNA Helix upon Treatment with Anthracycline Drugs
Source: Int J Mol Sci. 2020 Jun 10;21(11):4142. doi: 10.3390/ijms21114142 (PMC7312087; doi:10.3390/ijms21114142)
Supplement: Supplementary file 1 [file ijms-21-04142-s001.pdf]

Table S1 The comparison of the length of DNA chains before and after interaction with anthracycline antibiotics

	**DNA lenght [nm]**		
	DNA 35% GC	DNA 52% GC	DNA 77% GC
**Control**	144.76 ± 10.30	147.81 ± 6.80	147.57 ± 7.48
**DOX 0.3 μM**	154.23 ± 6.88	155.56 ± 8.61	172.22 ± 6.79
**DOX 2.0 μM**	191.48 ± 16.06	195.21 ± 12.01	228.84 ± 15.79
**DAU 0.3 μM**	151.58 ± 6.68	157.21 ± 6.29	173.58 ± 12.44
**DAU 2.0 μM**	157.30 ± 8.86	161.91 ± 5.72	174.34 ± 16.57
**EPI 0.3 μM**	155.68 ± 10.81	171.35 ± 8.97	172.94 ± 13.96
**EPI 2.0 μM**	176.70 ± 13.53	192.24 ± 8.92	196.53 ± 6.53

Table S2 The comparison of the height of DNA chains before and after interaction with anthracycline antibiotics

	**DNA height [nm]**		
	**DNA 35% GC**	**DNA 52% GC**	**DNA 77% GC**
**Control**	1.044 ± 0.1044	1.013 ± 0.071	1.012 ± 0.068
**DOX 0.3 μM**	0.739 ± 0.078	0.615 ± 0.057	0.578 ± 0.066
**DOX 2.0 μM**	0.489 ± 0.046	0.456 ± 0.042	0.342 ± 0.033
**DAU 0.3 μM**	0.801 ± 0.057	0.690 ± 0.070	0.556 ± 0.084
**DAU 2.0 μM**	0.637 ± 0.027	0.598 ± 0.057	0.526 ± 0.052
**EPI 0.3 μM**	0.705 ± 0.040	0.651 ± 0.075	0.557 ± 0.055
**EPI 2.0 μM**	0.508 ± 0.045	0.480 ± 0.036	0.447 ± 0.052

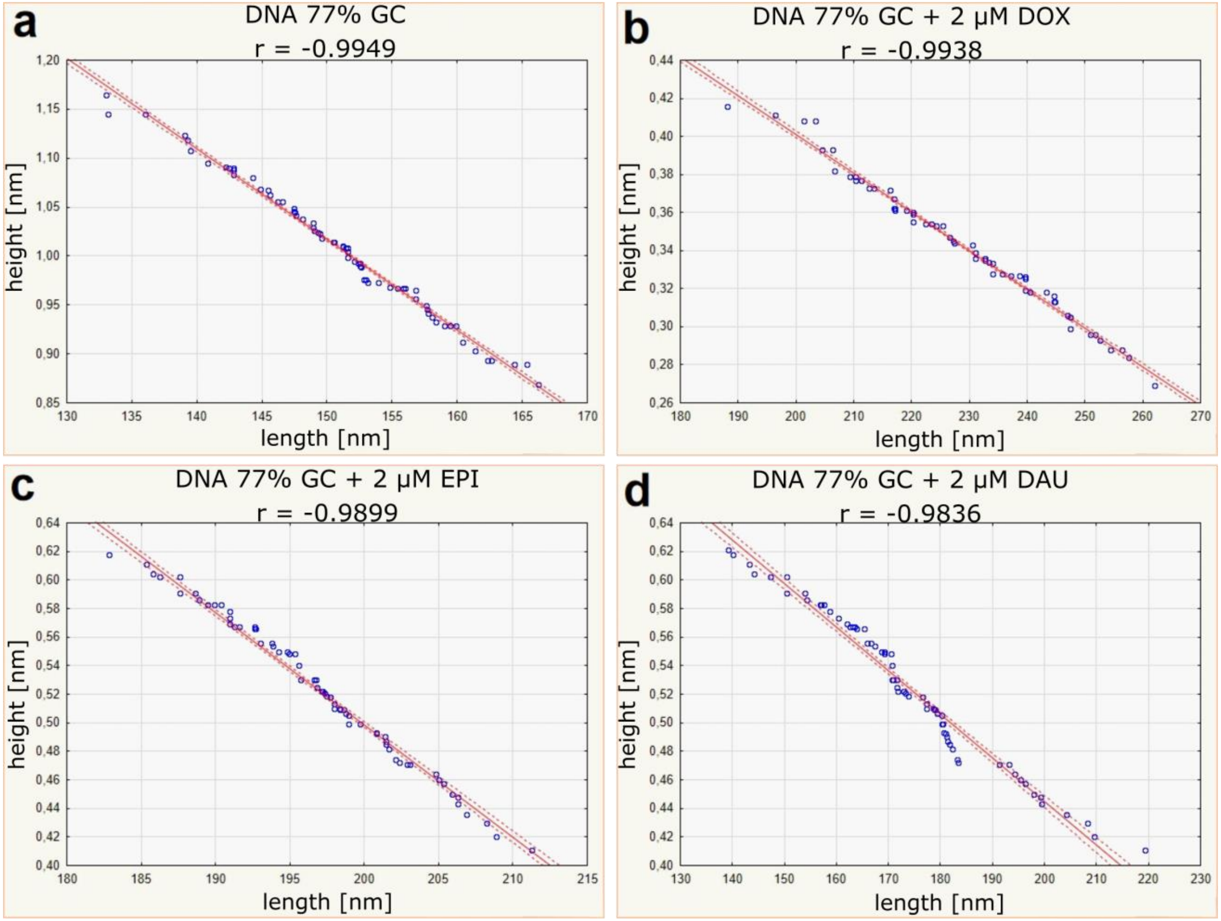

Figure S1 Linear correlation between the length and height of the DNA strand without drug (a) and after interaction with 2 μM DOX (b), 2 μM EPI (c) and 2 μM DAU (d)
